# Exploring Co-Occurrence Patterns to Understand Epiphyte–Liana Interactions

**DOI:** 10.3390/plants14010140

**Published:** 2025-01-06

**Authors:** Sergio J. Ceballos, Ezequiel Aráoz, Tobías Nicolás Rojas

**Affiliations:** 1 Instituto de Ecología Regional (IER), Universidad Nacional de Tucumán (UNT)-Consejo Nacional de Investigaciones Científicas y Técnicas (CONICET), Yerba Buena 4107, Tucumán, Argentina; ezequielaraoz@gmail.com (E.A.); tobiasnrojas@gmail.com (T.N.R.); 2 Facultad de Ciencias Naturales e Instituto Miguel Lillo, Universidad Nacional de Tucumán, San Miguel de Tucumán 4000, Tucumán, Argentina; 3 Mueller Laboratory, Biology Department, The Pennsylvania State University, University Park, PA 16802, USA

**Keywords:** canopy ecology, competition, species interactions, trees, Yungas

## Abstract

Although epiphytes and lianas share the same habitat, most research has treated these two groups independently. This study aimed to evaluate the co-occurrence of vascular epiphytes and lianas in the subtropical montane forests of northwestern Argentina. We recorded epiphyte cover and liana basal area on trees ≥ 10-cm-dbh in 120 20 × 20 m plots in the Sierra de San Javier (Tucumán, Argentina). Of the 2111 trees sampled, 727 (34%) hosted lianas, and 1095 (52%) hosted epiphytes. Both plant groups were found together on 20% of the sampled trees. The species richness of lianas and epiphytes, along with liana basal area and epiphyte cover, increased with tree diameter and reached higher values in mature forests compared to successional forests. Both groups colonized the same canopy tree species with larger diameters, whereas smaller trees were typically colonized by either lianas or epiphytes, but not both. Epiphyte species were more likely to co-occur with liana species with specialized climbing mechanisms. Tree size and forest type (mature vs. successional) emerged as key factors influencing the co-occurrence of lianas and epiphytes in these forests. This study establishes a basis for future research into the interactions between lianas and epiphytes, seeking to determine whether they co-occur in the same habitats.

## 1. Introduction

Trees have outstanding ecological value in supporting the biodiversity of the forest canopy [1,2]. In particular, for lianas and epiphytes, trees play a fundamental role, by providing support and a diversity of microhabitats [3,4]. Lianas climb tree trunks in search of light, while epiphytes germinate and root on trees without parasitizing them [2,5,6,7]. Although both plant groups rely on trees for structural support, our understanding of how lianas and epiphytes interact remains limited. Despite sharing the same trees, most research has examined these plant groups separately, focusing on liana–tree or epiphyte–tree relationships [2,8,9]. Consequently, little is known about whether lianas and epiphytes compete against or facilitate each other on shared trees.

Lianas and epiphytes exhibit temporal segregation during forest succession. In tropical forests, the abundance and richness of these two plant groups increase at different stages of succession in a predictable pattern [4,10]. Specifically, in forests regenerating from abandoned agricultural fields, pastures, or logged areas, lianas colonize rapidly during the first 30 years of forest recovery [10,11,12,13]. Lianas favor the conditions typical of early successional forests, such as high light availability, low canopy height, and the abundance of small-diameter trees suitable for climbing [10,11]. As forests mature, liana abundance decreases, while epiphyte abundance increases [14,15,16]. Epiphytes colonize more slowly during succession, becoming more prevalent in older forests where large-diameter trees are more common [4,17,18]. These larger trees provide more surface, microhabitats, and longer time exposure for epiphyte colonization [9,17,19].

In addition to temporal segregation, lianas and epiphytes may also segregate spatially due to differences in their microhabitat niches [20,21]. For instance, hemiepiphytes have been observed to thrive better in moist sites compared to lianas [21,22,23]. Conversely, lianas often form dense clumps around trees at the edges of dry forests, producing large amounts of foliage above their hosts and leaving limited space for epiphytic plants [20]. Interactions with other organisms could also contribute to the segregation of lianas and epiphytes. For example, in a Bornean tropical rainforest, a species of ant that forms symbiotic relationships with two epiphytic ferns actively prunes and removes lianas [24]. However, research remains limited, and general mechanisms explaining the interactions between lianas and epiphytes beyond their temporal and spatial segregation are yet to be established.

As in other regions, lianas and epiphytes in the subtropical montane forests of Argentina have been studied separately [25,26]. Research in this area has demonstrated that both lianas and epiphytes are significant components of biodiversity, particularly abundant in mature forests [9,27]. A study performed in these forests showed that large and well-lit trees were more likely to support lianas and hosted a greater liana abundance than small and shaded trees [27]. Similarly, another study revealed that larger-diameter trees supported more vascular epiphyte species than smaller-diameter trees in the same area [9]. Additionally, the interactions between these plant groups and host tree characteristics have been analyzed, but only in mature forests [9,27]. Given that both groups are abundant in similar habitats, these subtropical forests provide an excellent setting for investigating their interactions.

To better understand the interactions between lianas and epiphytes, it is important to determine whether they share the same host tree species and coexist on the same trees within the same forests. In this study, we combined two datasets of lianas and epiphytes collected from the same mature and successional forest plots as an initial step to investigate their interactions. The objective of this study was to assess the co-occurrence of vascular epiphytes and lianas—plants that depend on trees for support—in the subtropical montane forests of northwestern Argentina. Specifically, we aimed to carry out the following actions: 1. Compare the occurrence of lianas and epiphytes at three ecological scales (i.e., tree zone, tree, and forest patch). 2. Test whether forest type (i.e., successional vs. mature) or tree size explains the species richness of lianas and epiphytes, liana basal area, and epiphyte cover. 3. Explore co-occurrence patterns among liana, epiphyte, and tree species using interaction networks. 4. Analyze the spatial association of trees hosting lianas and epiphytes. We expected the following outcomes: 1. Lianas and epiphytes will coexist on the same trees and in the same areas of each tree, though their occurrence will vary across different forest patches. 2. Since both lianas and epiphytes require space on trees to grow, the richness and abundance of these groups will increase on large-diameter trees in mature forests. 3. Lianas and epiphytes will interact with the same set of tree species. 4. Trees hosting lianas will be spatially associated with those hosting epiphytes.

## 2. Materials and Methods

### 2.1. Study Area

**Figure 1:**
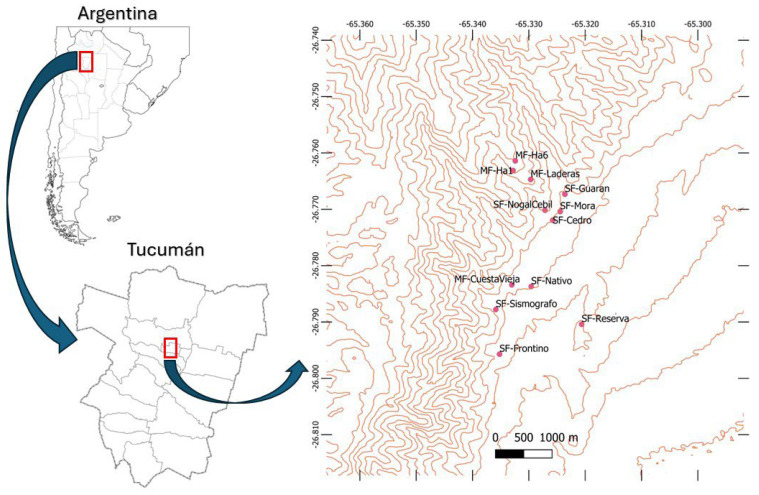
Distribution of the 12 forest patches in the Sierra de San Javier, Tucumán, Argentina. SF and MF are successional and mature forests, respectively. Red boxes indicate the detailed areas shown in the subsequent sub-figure.

We conducted this study in Parque Sierra de San Javier (26°46′3.5″ S, 65°29′22.5″ W), a 14,000 ha protected area located between 600 and 1800 m asl in Tucumán, northwest Argentina (Figure 1). The area lies 13 km west of Gran San Miguel de Tucumán, a city of approximately one million inhabitants, whose influence has shaped the land cover and forest dynamics of Sierra de San Javier over the past centuries [28]. Historically, the main activities in the Sierra included agriculture and livestock grazing, which replaced previously forested areas. However, the establishment of the protected area in 1973, along with rural outmigration and reduced land use intensity, has transformed the landscape into a mosaic of shrublands and successional forests [28,29].

The study area has a subtropical climate characterized by a pronounced dry season from April to October and occasional winter frosts [30]. Mean annual precipitation ranges from 1300 to 1500 mm, and the mean annual temperature is 18 °C [31].

The vegetation corresponds to subtropical montane forests, representing the southernmost extension of Neotropical Andean forests [32]. This study focused on low mountain forests (700–1000 m asl), including mature and successional forests. Mature forests are dominated by tree species such as *Blepharocalyx salicifolius*, *Ocotea porphyria*, and *Pisonia zapallo*, while the subcanopy is primarily composed of *Eugenia uniflora*, *Piper tucumanum*, and *Allophylus edulis* [27,33]. Successional forests, which have developed mainly on abandoned croplands, contain native tree species such as *Tecoma stans*, *Heliocarpus popayanensis*, *Parapiptadenia excelsa* and *Tipuana tipu*, along with exotic tree species such as *Ligustrum lucidum* and *Morus alba* [29].

In the Sierra de San Javier, both lianas and epiphytes are abundant and diverse in the Yungas forests [34,35]. They occur on approximately 62–65% of trees with a diameter greater than 10 cm [25,26,27]. In the area, mature forests provide optimal habitats for lianas and epiphytes due to the presence of treefall gaps and large-diameter trees [34,36]. However, these plants are also present in successional forests that have regenerated from abandoned lands that were once used for farming and cattle grazing. The most abundant liana species are *Cissus striata*, *Celtis iguanaea*, and *Chamissoa altissima* [37]. The most common epiphyte species are *Pleopeltis tweediana*, *Microgramma squamulosa*, and *Aechmea distichantha* [34].

### 2.2. Data Collection

For this study, we considered the following definitions for lianas and epiphytes. Lianas are climbing plants with true wood or persistent, fibrous stems, which germinate on the ground and rely on external physical support to ascend to the canopy [38]. Epiphytes are plants that germinate and root non-parasitically on other plants [2]. The study area lacks hemiepiphytes and nomadic vines, so there was no ambiguity in distinguishing between lianas and epiphytes.

Lianas, vascular epiphytes, and trees were surveyed in 12 forest patches located at elevations between 700 and 1000 m above sea level in Parque Sierra de San Javier (Figure 1). Our study included eight successional forest patches, along with four mature forests that did not show recent signs of human disturbance [29,34,35].

In every forest patch we sampled ten 20 × 20 m plots. Most plots were spaced 20 m apart and located more than 10 m from the forest edge. An exception was one forest patch (“Cedro”, Figure 1), which included nine contiguous plots (0.36 ha) monitored every 5 years by the Red Subtropical de Parcelas Permanentes (RedSPP) (https://ier.conicet.gov.ar/red-subtropical-de-parcelas-permanentes-redspp/ (accessed on 28 November 2024)) and one additional plot located 20 m away. Within each plot, we identified and measured the diameter at breast height (dbh) of all trees ≥ 10 cm. In the Cedro forest patch, we also mapped the location of each tree using an x–y coordinate system to perform spatial analyses in the nine contiguous plots.

In each plot, we identified and measured the diameter of lianas supported by trees, focusing on woody climbers with a diameter of ≥1 cm at 130 cm from the main rooting point [38]. Additionally, we identified vascular epiphyte species and estimated their total cover per tree using binoculars from the ground. We acknowledge that ground-based observations may underestimate the richness and frequency of epiphytes [39]. However, we selected this method to sample a large number of trees in an area with relatively low epiphyte richness [26]. Cover was estimated using a modified Braun-Blanquet scale, considering the percentage of the tree surface covered by epiphytes (1 = 1 to 5%, 2 = 6 to 25%, 3 = 26 to 50%, 4 = 51 to 75%, and 5 = 76 to 100%) [40]. Additionally, we recorded the number of occurrences of lianas and epiphytes on the crowns and trunks of the trees.

### 2.3. Data Analysis

We compared the occurrence of lianas and epiphytes across three ecological scales: tree zone, tree, and forest (objective 1). At the tree zone scale, we compared the frequency of occurrence of lianas and epiphytes between the trunk and crown of trees using generalized linear models (GLMs) with a negative binomial distribution. At the tree scale, we used Chi-square tests to analyze the associations between the presence and absence of lianas and epiphytes in trees of four different size classes. At the forest scale, we compared the number of trees colonized by lianas or epiphytes across 12 forest patches using generalized linear models (GLMs) with a negative binomial distribution.

To analyze the effects of forest type (i.e., successional or mature) and tree size (dbh) on the richness of lianas and epiphytes, liana basal area, and epiphyte cover, we performed four Bayesian mixed-effects linear regressions (objective 2). In these models, forest type and tree size were the explanatory variables, and tree species was set as a random variable. This way, we developed a model for each response variable (i.e., liana basal area, liana richness, epiphyte richness, and epiphyte cover). Liana abundance posteriors were estimated using a log-normal hurdle distribution, due to the high number of zeros in the sampled trees. For the abundance of epiphytes, we used cumulative distribution, since the response variable is ordinal. For the richness of lianas and epiphytes, we used a hurdle Poisson distribution. In all cases, we ran four Markov chains of 8000 iterations each. The priors were weakly informative in each of the four models (Supplementary Materials S1). All models converged successfully and reached acceptable Rhat and effective sample size (ESS) values (Rhat < 1.01 and ESS > 1000; Supplementary Materials S1).

We explored the relationships between liana and tree species, epiphytes and trees, and lianas and epiphytes using interaction networks (objective 3). To describe the frequency of interactions between lianas and trees, we considered the total basal area of liana species associated with each tree species. For the epiphyte–tree interaction network, we described the frequency of interactions based on the number of zones [41] occupied by each epiphyte species on each tree species. For the liana–epiphyte interaction network, we only included trees where both lianas and epiphytes occurred together. All interaction networks were generated using the bipartite library in R [42]. In addition, we compared the number of epiphyte co-occurrences with lianas that use specialized climbing mechanisms versus those that merely lean on tree branches, using a generalized linear model with a negative binomial distribution.

We conducted a point pattern analysis to investigate the spatial association of trees hosting lianas or epiphytes, at different distances (objective 4). This analysis was carried out within the 0.36 ha permanent plot (“Cedro”), where the trees were mapped using an x- and y-coordinate system. The input for our analyses included tree locations, as well as the presence of lianas and epiphytes, as categorical marks. To assess these spatial patterns, we employed the K function, which examines the frequency of additional points (e.g., a tree) at increasing distances (radii) from each focal point. To reduce the effect of autocorrelation due to cumulative counts of additional points, we only considered points within two-meter-wide rings. To do so, the K value of each radius was recalculated by subtracting the K value corresponding to a two-meter-lower radius. We analyzed the spatial pattern of marked points (e.g., trees with lianas or epiphytes) and the frequency of crossed interactions (i.e., we evaluated potential interactions between lianas and epiphytes at different distances). We contrasted the observed pattern with a null model using Monte Carlo permutations. Specifically, we conducted 10,000 simulations to establish confidence intervals defined by maximum and minimum values at a significance level of α = 0.05. If the observed pattern falls within the confidence intervals, it suggests that the distribution of trees with lianas and epiphytes is consistent with the noninteraction hypothesis. Conversely, if the observed pattern deviates from these intervals, it indicates that the probability of finding a tree with epiphytes or lianas at different distances either increases or decreases. In the permutations, we considered the influence of tree size on the presence of lianas and epiphytes by adjusting binomial models, and used the predicted probabilities to reshuffle marks. Spatial analyses were performed using the spatstat package in R [43,44].

## 3. Results

### 3.1. Liana and Epiphyte Occurrences

In this study, we surveyed 2111 trees, of which 727 (34%) were hosts of lianas and 1095 (52%) of epiphytes. Of the total number of trees with lianas, only 44 trees (6%) had exclusively lianas and no epiphytes. In contrast, of the total number of trees with epiphytes, 62% hosted exclusively epiphytes and no lianas. Each tree had a median of one liana species (range: 1–5) and a mean basal area of 0.0025 square meters of lianas (range: 0.0001–0.0295). The trees had a median of two epiphyte species (range: 1–9) and a median epiphyte cover of 1 (range: 1–5).

**Figure 2:**
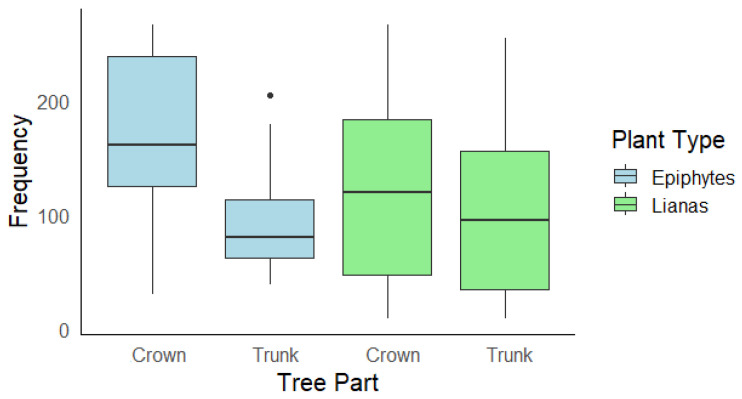
Number of occurrences of lianas and epiphytes on the crown and trunk of trees. The isolated point represents an outlier, a value beyond 1.5 times the interquartile range from the first and third quartiles.

At the tree zone scale, there were no significant differences between liana and epiphyte occurrences in the trunk (estimate = 0.06, z = 0.24, *p* = 0.81) and crown of trees (estimate = −0.31, z = −1.17, *p* = 0.24). The crown had a significantly higher occurrence of epiphytes compared to the trunk (estimate = −0.55, z = −2.72, *p* = 0.006; Figure 2). In contrast, there was no significant difference between the trunk and crown for liana occurrence (estimate = −0.18, z = −0.56, *p* = 0.57; Figure 2).

At the tree scale, the presence and absence of lianas and epiphytes were not significantly associated across four tree diameter classes (Table 1). Lianas and epiphytes shared few small trees (i.e., dbh < 13.15 cm), and almost half of the trees larger than 33.76 cm in dbh (Table 1). However, in both cases, there was no deviation from the expected proportions of lianas and epiphytes colonizing the trees.

On average, 20% of the trees hosted both lianas and epiphytes in most forest patches (Table 2). In certain forest patches, there was a significant prevalence of trees hosting epiphytes compared to the number of trees with lianas (e.g., Reserva, NogalCebil, Frontino, and CuestaVieja; Table 2 and Table 3). In contrast, in one of the forest patches (i.e., Mora), there were twice as many trees colonized by lianas as by epiphytes (Table 2 and Table 3).

### 3.2. The Influence of Forest Type and Tree Size on Lianas and Epiphytes

**Figure 3:**
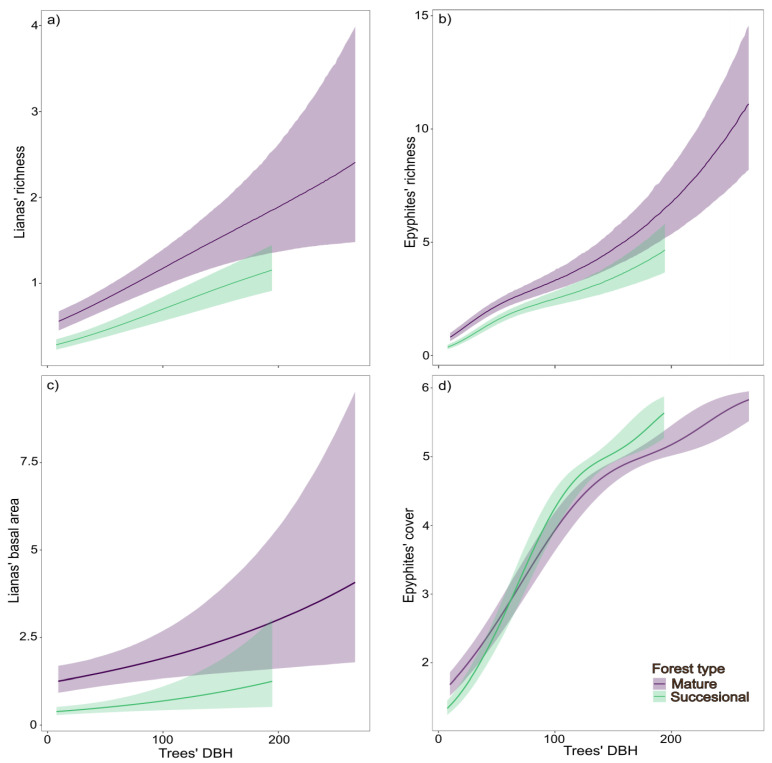
Comparison between mature and successional forests with respect to the association between trees´ dbh and (**a**) the species richness of lianas, (**b**) species richness of epyphites, (**c**) basal area of lianas and (**d**) epyphites´ cover.

The type of forest influenced liana basal area, epiphyte cover, and the species richness of both lianas and epiphytes in a similar way. Trees in mature forests tended to host more species of lianas and epiphytes compared to those in successional forests (Figure 3a,b). Similarly, tree size was positively associated with liana and epiphyte species richness, liana basal area, and epiphyte cover (Figure 3c,d). Details on the estimations are provided in Supplementary Materials S1.

### 3.3. Interaction Networks Between Lianas, Epiphytes, and Trees

**Figure 4:**
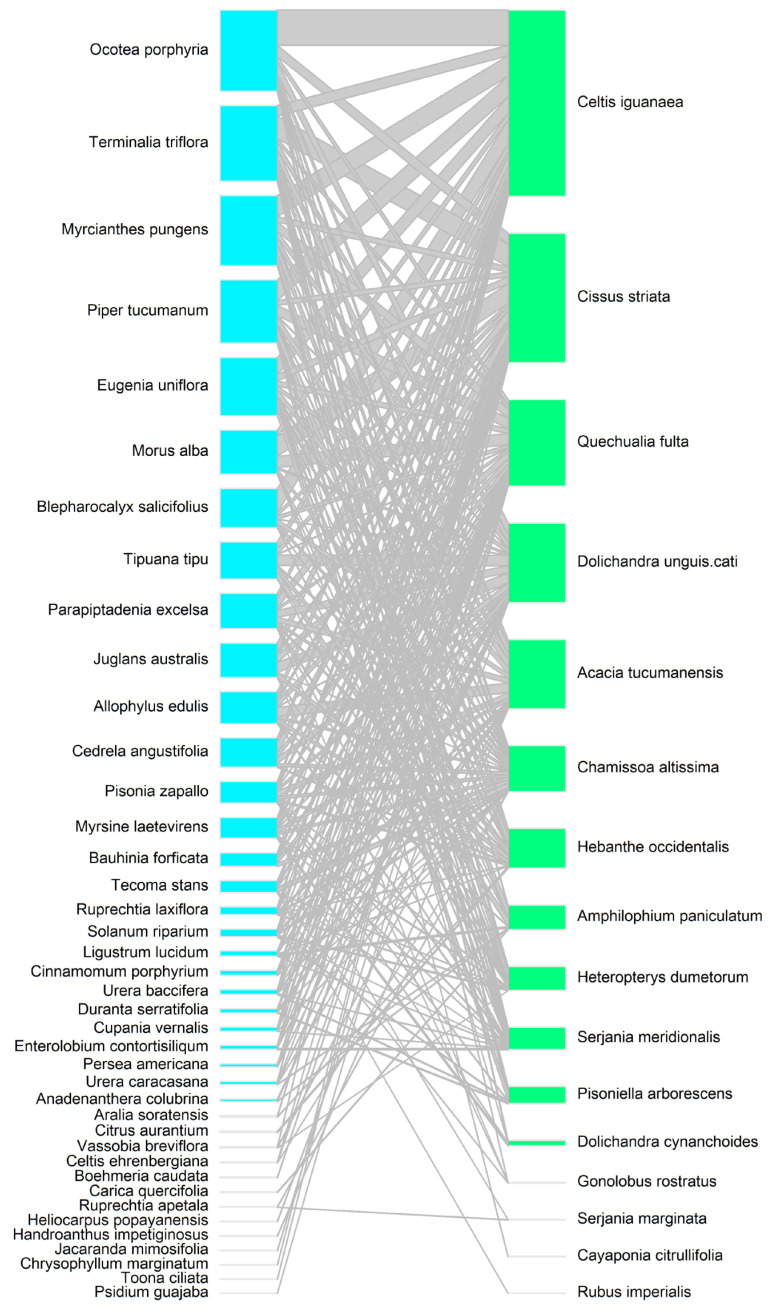
Interaction network between species of trees (**left**) and lianas (**right**). The size of the boxes represents the frequency of interactions between species.

**Figure 5:**
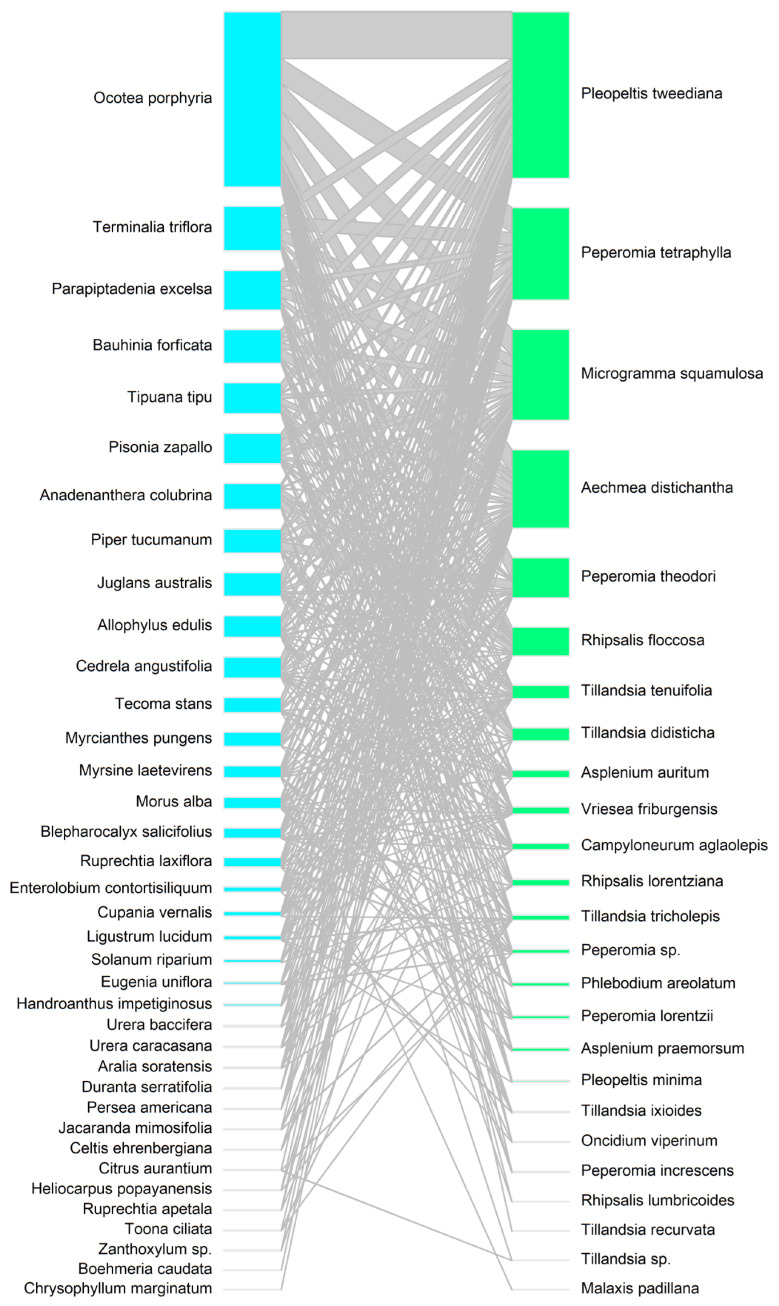
Interaction network between species of trees (**left**) and epiphytes (**right**). The size of the boxes represents the frequency of interactions between species.

**Figure 6:**
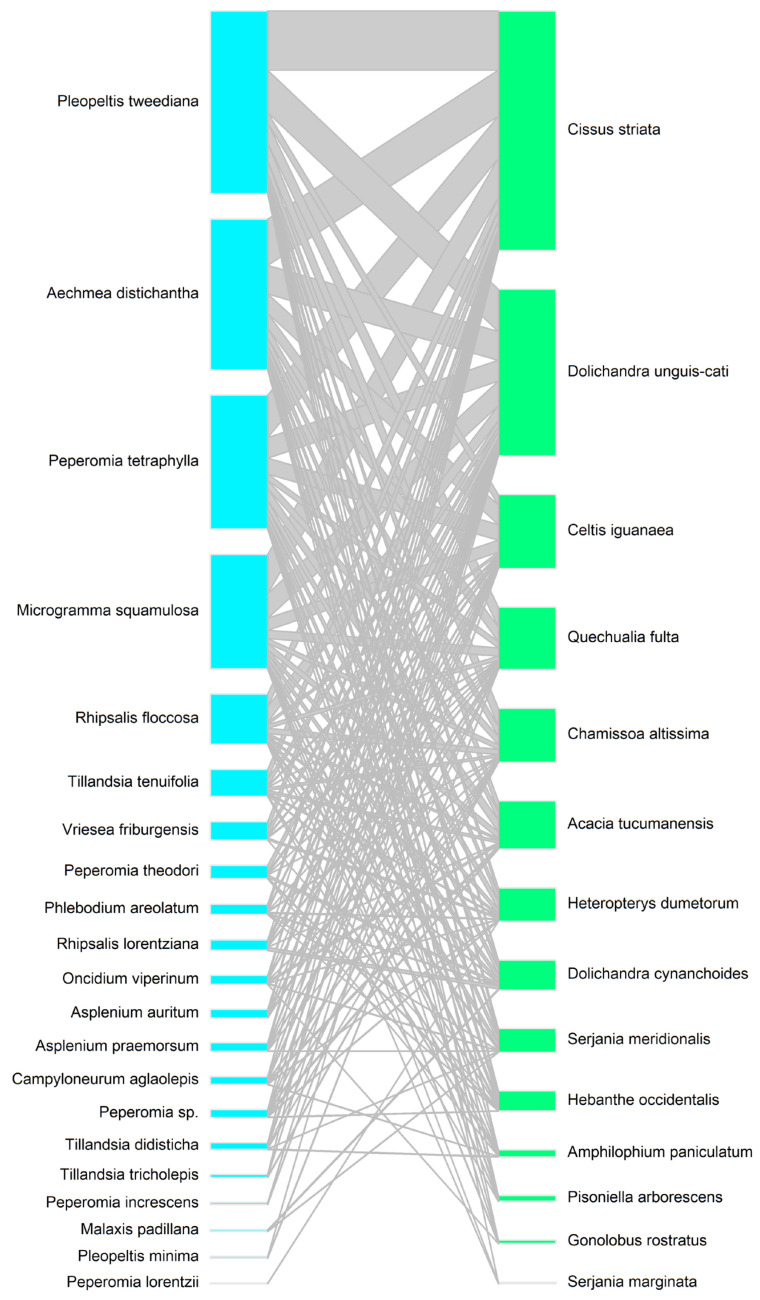
Interaction networks between species of epiphytes (**left**) and lianas (**right**). The size of the boxes represents the frequency of interactions between species.

In general, lianas and epiphytes interacted with the same tree species (Figure 4 and Figure 5). However, there were differences in the frequency of interactions that trees had with liana and epiphyte species. The liana species that showed the highest frequency of interaction with tree species were *Celtis iguanaea*, *Cissus striata*, *Quechualia fulta*, and *Dolichandra unguis-cati*. Liana species interacted most frequently with canopy tree species such as *Ocotea porphyria* and *Terminalia triflora*, as well as with species in the middle and lower strata, such as *Myrcianthes pungens*, *Piper tucumanum*, *Eugenia uniflora*, and *Morus alba* (Figure 4). In contrast, epiphytes interacted almost exclusively with canopy trees such as *Ocotea porphyria*, *Terminalia triflora*, and *Parapiptadenia excelsa*. *Ocotea porphyria* was the tree species that had the most interactions with epiphyte species, including *Pleopeltis tweediana*, *Peperomia tetraphylla*, *Microgramma squamulosa*, *Aechmea distichantha*, and *Peperomia theodori* (Figure 5). Among the epiphyte–liana interaction network, epiphyte species co-occurred most frequently with *Cissus striata* and *Dolichandra unguis-cati* (Figure 6). There were significantly more epiphyte occurrences on trees with liana species that use specialized climbing mechanisms than on trees with lianas that merely lean on tree branches (estimate = 1.21, z = 2.74, *p* = 0.006).

### 3.4. Spatial Patterns of Trees Hosting Lianas and Epiphytes

**Figure 7:**
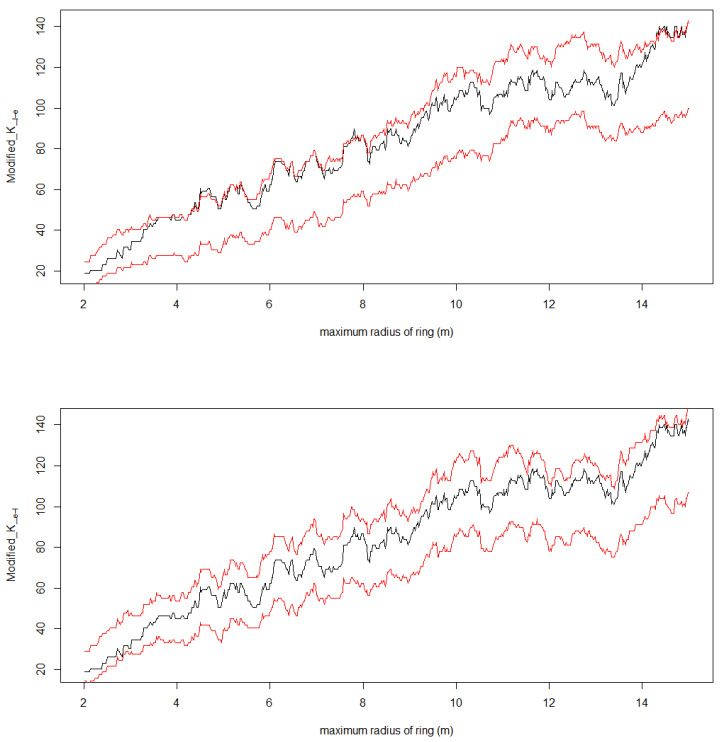
Spatial arrangement of trees with lianas and epiphytes. The curves in black indicate the pair of epiphytes and lianas at increasing distances. The confidence intervals in red indicate the central 95% values estimated from 10,000 permutations. In each of these permutations, we randomly distributed epiphytes (**above**) and lianas (**below**) considering the likelihood of a tree being colonized given its species identity and dbh.

The contiguous 0.36 ha plot contained 120 trees; 66 trees hosted at least one liana and 39 hosted at least one epiphyte. Point pattern analysis revealed that the likelihood of encountering a tree with epiphytes at 3 to 7 m from a tree with lianas was slightly higher than expected, by chance only when the host species and dbh were taken into account (Figure 7). By contrast, the likelihood of a tree hosting lianas did not change in response to an epiphyte-hosting tree at any distance (Figure 7).

## 4. Discussion

In this study, we evaluated the co-occurrence of lianas and epiphytes in the subtropical montane forests of northwestern Argentina as an initial step to investigate their potential interactions. To date, lianas and epiphytes have been studied separately, making this the first study to analyze both groups together in this region. We combined two databases of lianas and epiphytes surveyed in the same forest plots to explore patterns of occurrence at different ecological scales (tree zone, tree, and forest patch). The results showed that both lianas and epiphytes occur on the trunk and crown of trees, sharing, on average, 20% of the host trees within the same plots. However, the proportion of shared trees varied considerably between plots (3.3–52.5%), suggesting that local conditions may favor one group over the other [34,35].

The forest structure, composition, and successional status of forest patches may influence the varying prevalence of lianas and epiphytes. In particular, the forest patches Reserva, NogalCebil, Frontino, and CuestaVieja showed a higher proportion of trees with epiphytes compared to those hosting lianas. These forest patches are characterized by the dominance of one or two species of trees, such as *Anadenanthera colubrina* in “Reserva”, *Juglans australis* and *Parapiptadenia excelsa* in “NogalCebil”, *Ocotea porphyria* in “Frontino”, and *Bauhinia forficata* in “CuestaVieja”, which possess characteristics favorable for epiphyte colonization [26,34,45]. In addition, these patches have a homogeneous structure, with a single cohort of tall trees that are difficult for many species of lianas to reach. In contrast, forests with lower canopy heights and the presence of fallen trees tend to harbor more lianas, as these conditions enhance light availability and facilitate climbing [7,8]. For instance, the “Mora” forest has a substantial number of fallen trees [28,46,47], resulting in a prevalence of trees with lianas over those with epiphytes. However, these remain merely hypotheses that should be evaluated in the future and take into account the factors that determine whether a forest supports more epiphytes than lianas, or vice versa.

Tree diameter was an important factor influencing the occurrence of lianas and epiphytes. In smaller trees, co-occurrence was low, as they were generally colonized by either lianas or epiphytes, but not both. Nearly 50% of trees with a diameter greater than or equal to 33.8 cm were simultaneously colonized by lianas and epiphytes. In addition, tree diameter was positively associated with liana and epiphyte species richness, liana basal area, and epiphyte cover. This pattern could be explained by the size and age of the trees, as they offer a larger surface area and have been exposed to colonization by lianas and epiphytes for a longer time [9,26,27]. These findings align with results from other forests, showing that both epiphyte [17,19,48] and liana [49,50,51] prevalence increases among larger trees.

A higher species richness of lianas and epiphytes, as well as a greater liana basal area, was found in mature forests compared to successional forests. This pattern was expected for epiphytes but not for lianas, which tend to be more prevalent in successional stands [10,11,13,14,18]. These results underscore the conservation importance of mature forests, as they harbor the largest trees that support a high diversity of lianas and epiphytes. Moreover, mature forests are more stratified, allowing them to host liana species with diverse climbing modalities adapted to different tree sizes. However, this does not diminish the importance of successional forests for the conservation of these plants. It is essential to recognize that successional forests are in active recovery and, if protected from significant human impacts, could eventually achieve a diversity of lianas and epiphytes comparable to that of mature forests [10,18].

The presence of lianas and epiphytes was greater in large tree species that reach the canopy (e.g., *Ocotea porphyria*, *Terminalia triflora*, *Parapiptadenia excelsa*, and *Tipuana tipu*). On these tree species, epiphytes were found alongside liana species that had specialized climbing mechanisms to reach the canopy. For example, epiphyte species were commonly found in trees hosting lianas with tendrils and adventitious roots (e.g., *Dolichandra unguis-cati* and *Cissus striata*), as well as those with spines (*Celtis iguanaea* and *Acacia tucumanensis*) [36]. On the other hand, liana species that merely lean on tree branches (e.g., *Quechualia fulta* and *Chamissoa altissima*) were more frequent in smaller trees (e.g., *Eugenia uniflora* and *Piper tucumanum*), which are typically not colonized by epiphytes [9]. Therefore, epiphytes could be spatially segregated from certain liana species due to the biology of lianas (i.e., specifically their ability to climb trees of certain sizes) rather than competition.

Other studies suggested that lianas could have a negative influence on epiphytes, as lianas cover host trees with a large number of leaves, leaving little space available for epiphytes [20,21]. It has also been suggested that in successional forests, abundant lianas may outcompete epiphytes by increasing shade and occupying colonization sites [52]. In this study, specifically in a successional forest highly dominated by lianas, we found a slight effect of the distance from liana-hosting trees on the likelihood of finding an epiphyte. However, caution is necessary when interpreting this as an effect of lianas on epiphytes, as the effect was not very strong. Furthermore, the non-replicated design and small plot size (0.36 ha) may have limited our ability to detect a conclusive pattern regarding the interactions between lianas and epiphytes.

In this study, the occurrence patterns of lianas and epiphytes were analyzed as an initial approach to understanding the interactions between these two groups of plants. The results indicate that, in this region, both lianas and epiphytes tend to co-occur primarily in mature forests and on larger-diameter trees. Furthermore, it was observed that epiphytes do not coexist uniformly with all liana species, but predominantly with those that have specialized climbing mechanisms. To further explore the nature of these interactions, future research should consider the patterns identified in this study. For instance, studies investigating the type of interaction between lianas and epiphytes in this region should focus on: (1) forests with high cover or abundance of both life forms, (2) tree species commonly hosting both lianas and epiphytes, and (3) specific interactions between epiphytes and certain liana species. In addition, it would be necessary to use a common variable for both lianas and epiphytes, such as canopy or bark occupancy. Building on these aspects, we can further investigate whether lianas and epiphytes compete for the same habitat or facilitate each other’s presence.

## Figures and Tables

**Table 1:** Number of trees hosting lianas and epiphytes per dbh class.

Tree dbh Quartiles	No. of Trees	No. of Trees Hosting Lianas	No. of Trees Hosting Epiphytes	No. of Trees Shared Between Lianas and Epiphytes	Chi-Square Test (*p*-Value)
1 (<13.15 cm)	528	123	130	27 (11.9%)	0.44 (0.51)
2 (>13.15–<19.75)	535	156	223	62 (19.6%)	0.24 (0.62)
3 (>19.75–<33.76)	521	178	319	111 (28.8%)	0.08 (0.77)
4 (>33.76)	527	270	423	215 (45%)	0.07 (0.79)

**Table 2:** Number of trees hosting lianas and epiphytes per forest patch.

Forest Patch Label	Forest Type	No. of Trees	No. of Trees Hosting Lianas	No. of Trees Hosting Epiphytes	No. of Trees Shared Between Lianas and Epiphytes
Guarán	Successional	197	91	110	60 (42.6%)
Nativo	Successional	182	33	49	17 (26.2%)
Reserva	Successional	226	7	120	4 (3.3%)
Mora	Successional	141	82	43	32 (34.4%)
NogalCebil	Successional	146	24	95	21 (21.4%)
Cedro	Successional	147	73	55	37 (40.7%)
Sismógrafo	Successional	173	53	61	19 (20%)
Frontino	Successional	168	32	134	27 (19.4%)
CuestaVieja	Mature	190	63	147	47 (28.8%)
Laderas	Mature	135	61	90	52 (52.5%)
Ha1	Mature	210	105	102	54 (35.3%)
Ha6	Mature	196	103	86	45 (31.3%)

**Table 3:** Results of generalized linear models comparing the number of trees colonized by lianas or epiphytes per forest patch. * indicate that differences are statistically significant.

Forest Patch Label	Estimate	z Value	*p* Value
Guarán	−0.18	−0.85	0.39
Nativo	−0.39	−1.10	0.27
Reserva	−2.84	−7.31	0.001 *
Mora	0.64	2.88	0.003 *
NogalCebil	−1.37	−5.03	0.001 *
Cedro	0.28	1.02	0.31
Sismógrafo	−0.14	−0.58	0.55
Frontino	−1.43	−7.27	0.001 *
CuestaVieja	−0.84	−4.15	0.001 *
Laderas	−0.38	−2.34	0.09
Ha1	0.01	0.13	0.89
Ha6	0.18	1.20	0.22

## Data Availability

Data are available at this link: http://hdl.handle.net/11336/250932 (accessed on 28 November 2024).

## Supplementary Materials

The following supporting information can be downloaded at: https://www.mdpi.com/article/10.3390/plants14010140/s1, Supplementary Materials S1. BGLMM results.
