# Supplementary material for: Exploring Co-Occurrence Patterns to Understand Epiphyte–Liana Interactions
Source: Plants (Basel). 2025 Jan 6;14(1):140. doi: 10.3390/plants14010140 (PMC11722571; doi:10.3390/plants14010140)
Supplement: Supplementary file 1 [file plants-14-00140-s001.zip › plants-3370455-supplementary.pdf]

Supplementary Material S1. BGLMM results.

	Estimate	2.5% CI	97.5% CI	Rhat	ESS
Intercept 1	-0.875	-1.266	-0.469	1.00	3578
Intercept 2	1.124	0.726	1.537	1.00	3670
Intercept 3	2.192	1.781	2.614	1.00	3934
Intercept 4	3.805	3.338	4.289	1.00	4807
Intercept 5	8.936	7.553	10.527	1.00	15424
DBH	1.025	0.907	1.146	1.00	18848
Successional forest	-1.015	-1.254	-0.778	1.00	17278

Table S1. BGLMM results for tree DBH and forest type explaining epiphytes cover. Due to the ordinal nature of the response variable we set cumulative distribution with weakly informative priors. Flat for intercepts and t-student (3, 0, 2.5) for betas. Iterations of MCMC was set at 8000 and posterior estimation was done after burning the first 2000 values.

	Estimate	2.5% CI	97.5% CI	Rhat	ESS
Intercept	0.574	0.426	0.711	1.00	9659
Hurdle Intercept	-0.783	-1.188	-0.365	1.00	5727
DBH	0.174	0.147	0.201	1.00	31123
Successional Forest	-0.342	-0.468	-0.215	1.00	24742
Hurdle DBH	-1.712	-1.958	-1.477	1.00	32609
Hurdle successional forest	0.854	0.571	1.141	1.00	25934

Table S2. BGLMM results for tree DBH and forest type explaining epiphytes richness. We estimated a hurdle Poisson model due the high incidence of zeros in the data. We set flat prior for the intercept, beta (1,1) for the hurdle part of the model and t-student (3, 0, 2.5) for betas. Iterations of MCMC was set at 8000 and posterior estimation was done after burning the first 2000 values.

	Estimate	2.5% CI	97.5% CI	Rhat	ESS
Intercept	-6.410	-6.618	-6.206	1.00	10589
Hurdle Intercept	0.278	-0.023	0.600	1.00	6254
DBH	0.107	0.026	0.188	1.00	24720
Successional Forest	-0.742	-0.953	-0.524	1.00	19156
Hurdle DBH	-0.381	-0.499	-0.269	1.00	26577
Hurdle successional forest	0.673	0.421	0.925	1.00	20815

Table S3. BGLMM results for tree DBH and forest type explaining liana basal area. We estimated a hurdle log-normal model due the high incidence of zeros in the data. We set flat prior for the

intercept, beta (1,1) for the hurdle part of the model and t-student (3, 0, 2.5) for betas. Iterations of MCMC was set at 8000 and posterior estimation was done after burning the first 2000 values.

	estimate	2.5% CI	97.5% CI	Rhat	ESS
Intercept	-0.125	-0.327	0.055	1.00	20325
Hurdle Intercept	0.278	-0.032	0.588	1.00	8848
DBH	0.080	0.002	0.150	1.00	33261
Succesional Forest	-0.868	-1.149	-0.58’	1.00	25883
Hurdle DBH	-0.381	-0.501	-0.267	1.00	35227
Hurdle successional forest	0.670	0.417	0.920	1.00	25558

Table S4. BGLMM results for tree DBH and forest type explaining liana richness. We estimated a hurdle Poisson model due the high incidence of zeros in the data. We set flat prior for the intercept, beta (1,1) for the hurdle part of the model and t-student (3, 0, 2.5) for betas. Iterations of MCMC was set at 8000 and posterior estimation was done after burning the first 2000 values.
